# Psychosocial dental impact in adult orthodontic patients: what about health competence?

**DOI:** 10.1186/s12955-019-1179-9

**Published:** 2019-06-26

**Authors:** María José González, Martín Romero, Cecilia Peñacoba

**Affiliations:** 1 Department of Orthodontics, Universidad Rey Juan Carlos, Avda de Atenas s/n 28922 Alcorcón, Madrid, Spain; 2 Psychology Department, Rey Juan Carlos University, Alcorcón, Madrid, Spain

**Keywords:** Malocclusion, Oral health, Quality of life, Dental care, Esthetics, Outcome assessment

## Abstract

**Background:**

Several studies have assessed the psychological benefits of orthodontic treatment; however, the impact of competence on psychological benefits remains unknown.

Aims: To analyze the change of the perception of psychosocial dental impact in a sample of adults undergoing orthodontic treatment (mild/moderate dental malocclusions) and to assess the possible moderating effect of health competence level.

**Methods:**

A longitudinal prospective design was used. Three time points were included: baseline (T0), 6 months after starting orthodontic treatment (T1) and once treatment had finished (T2). The pretreatment sample consisted of 78 patients recruited from the Rey Juan Carlos University Dental Clinic, all of whom had moderate malocclusions and were going to undergo orthodontic treatment for approximately 18 months with fixed metal multibrackets. All participants were instructed to complete the Spanish version of the Psychosocial Impact of Dental Aesthetics Questionnaire (PIDAQ) and the aesthetic component of the Index of Orthodontic Treatment Need (IOTN-AC) on the three points of the research. Statistical analysis involved the General Linear Model (GLM) repeated-measures ANOVA to test if the outcome measures of psychosocial dental impact significantly changed over time during orthodontic treatment (baseline, at 6-month evaluation and posttreatment). To assess the effect of the previous health competence levels (high/low) in the change from baseline to the 6-month assessment, for each PIDAQ dimension, a 2*2 (time*group) repeated measures ANOVA was performed.

**Results:**

A significant increase was observed in dental self-confidence values (T0-T1 and T0-T2). Similar results were observed for the psychological impact variables and for the IOTN-AC scores, which showed significant decreases between T0 and T1 and between T0 and T2. Finally, significantly decreases were observed between T0 and T2 in aesthetic concern. Interaction effects were found regarding the health competence variable from T0-T1 for the psychological impact, social impact and aesthetic concern and the IOTN-AC index, with significant development results regarding the high competence group.

**Conclusions:**

The first 6 months of orthodontic treatment seemed to be key to the development of psychosocial dental impact perception, during which the role of health competence was of great importance to developing a positive change. It is necessary to follow a biopsychosocial approach towards orthodontic treatment.

## Introduction

Dental malocclusion in adults produces negative psychological impacts (difficulty chewing, pain, psychological discomfort as a result of dental problems and difficulty for social interaction), especially when the malocclusion is severe [1]. Several studies have assessed the psychological benefits of orthodontic treatment, taking into account oral health, quality of life (QoL) and the psychosocial impact of dental aesthetics as the main variables of study [2–8]. The above longitudinal studies differ in the number of time points. Some of them only include pre and posttreatment assessments [2, 3, 5, 8], while others include six measurements taken over time [4, 6, 7]. Even when the analyses of the results could be complicated due to the different methodologies used, in general, a short term change in the impact indicators can be observed, although that of the aesthetic concern is not entirely consistent. In the long term, all indicators experience a significant decrease.

Oral health-related quality of life (OHRQoL) has been defined as “the absence of negative impacts of oral conditions on social life and positive sense of dentofacial self-confidence” [9]. QoL in orthodontics has been analyzed in various age groups and for various severity levels of malocclusion (mild/moderate or severe malocclusion treated with orthognathic surgery) and orthodontic procedures (conventional/self-ligating brackets) [2–4, 10]. The results show that there are significant improvements in the QoL in patients with severe malocclusions undergoing treatment with orthognathic surgery, the most significant improvements being in psychological discomfort and disability domains [2]. In moderate malocclusions, improvements can also be observed in the physical pain and disability domains, although improvements in these patients are milder. The kind of brackets used (conventional metallic or self-ligating brackets) does not seem to be relevant to the improvement of the overall level of QoL [10].

There are few studies focusing on changes in psychosocial dental impact while patients are undergoing treatment.

During the orthodontic treatment, the psychosocial dental impact decreases, probably related to the aesthetic improvement perceived by the patient in the alignment of the anterior teeth [11]. This improvement has been observed at least 6 months after the beginning of treatment.

At the end of the orthodontic treatment, the patient’s perception of psychosocial dental impact in the dimensions such as dental confidence, social impact, psychological impact and aesthetic concern improves [5].

The development of psychosocial impact could be influenced by patient personality variables, some of which have been widely studied in health-disease processes, as is the case of internal control and self-perception competence [12]. Heath competence can be defined as “the degree to which an individual feels capable of effectively managing his or her health outcomes”. To date, no research studies have linked orthodontics and health competence.

In this context, the current study has two primary aims. The first is to analyze the change of the perception of psychosocial dental impact in a sample of adult patients undergoing orthodontic treatment. The second is to assess the possible moderating effect of health competence levels regarding the change of the perception of psychosocial dental impact. This paper has several practical implications for the clinician. The orthodontist could forecast the constraints that the patient will undergo through treatment and, based on that, improve the quality and effectiveness of patient care. On the other hand, the patient could benefit from this information to improve the adaptation process to treatment, as well as to increase active cooperation with the professional in achieving better treatment results.

To the best of our knowledge, there are no studies in the literature focusing on the changeof psychosocial dental impact during orthodontic therapy with conventional metal brackets in adults with mild/moderate malocclusion, which is one of the most frequent patient profiles in daily practice [13, 14].

## Materials and methods

### Design type

A longitudinal prospective design was used. Three time points were included: baseline, one day before bracket bonding (T0); 6 months after treatment began (T1), and bracket debonding (T2).

### Setting and sample

The study is part of a larger research project carried out at the Rey Juan Carlos University Dental Hospital, in South Madrid, Spain, which provides care to approximately 200 orthodontics patients per year. Adults registered for orthodontic treatment at this Dental Hospital were recruited between January 2015 and July 2016. Eligibility criteria were being over 18 years of age and having a mild/moderate dental malocclusions (molar class I, tooth size discrepancy lower than 3 mm and without a serious maxillary discrepancy). Patients were treated with fixed metal appliances over a period of 14 to 18 months. All patients were fluent in Spanish, so they could answer the proposed questionnaires. Two qualified orthodontists scored the sample using the IOTN-AC index (the aesthetic component of the IOTN index). Only patients scoring eight or above where ultimately included in our sample.

Exclusion criteria were having cognitive disorders, a history of previous orthodontic treatment, craniofacial deformities requiring orthognathic surgery, such as facial clefts syndromes, or being ineligible for orthodontic treatment due to unresolved dental pathologies (untreated dental caries, untreated tooth injury, or active periodontal disease).

The initial sample was calculated to provide 80% statistical power and 5% alpha in identifying a significant difference in psychosocial dental impact before and at the end of treatment (60 patients). Due to the expected experimental loss (approximately 30%), 78 patients were chosen and accepted to participate in the study; they signed informed consent and filled out the initial questionnaire (T0). At T1, 75 patients were followed-up to answer the questionnaire, and at T2, 71 patients participated (the nonresponses were due to missed appointments), achieving the proposed sample size.

### Instruments and measures

#### At three time points (T0, T1 and T2)

The Psychosocial Impact of Dental Aesthetics Questionnaire (PIDAQ) is one of many tools used to measure the impact of dental health on quality of life. Psychosocial dental impact perception was assessed using the Spanish version of the PIDAQ [15, 16]. The PIDAQ is a psychometric measure composed of 23 items divided into four subscales: dental self-confidence (6 items), social impact (8 items), psychological impact (6 items), and aesthetic concern (3 items). The first refers to positive perceptions, while the other three assess negative perceptions regarding various domains of psychosocial dental impact. Each item is scored on a five-point scale ranging from 0 to 4 (0 indicates “not at all”, 1 “a little”, 2 “somewhat”, 3 “strongly”, and 4 “very strongly”). The PIDAQ has been previously tested for its validity, reliability and factorial stability across samples [16–18]. Internal consistency in the sample for each of the dimensions was as follows: α = .90 for self-confidence, α = .86 for social impact, α = .88 for psychological impact, and α = .83 for aesthetic concern.

Orthodontic treatment need - aesthetic concern. To assess the self-perception of the severity of malocclusion, the aesthetic component of the Index for orthodontic treatment need-aesthetic concern (IOTN-AC) was used, as proposed by Brook and Shaw in 1989 [19]. The Aesthetic Component of the IOTN consists of a ten-point scale using ten photographs that relate to 10 possible degrees or levels of dental aesthetics. Higher scores indicate higher needs of treatment. The patient chooses which picture is more accurate in showing his or her own level of malocclusion.

#### At T0 (before treatment)

Perceived health competence. The Perceived Health Competence Scale (PHCS) was used [12]. It is a one-dimensional instrument composed of 8 items assessing beliefs regarding competence in facing health problems. Each item is scored on a five-point scale ranging from 0 to 4 (0 indicates “not at all”, 1 “a little”, 2 “somewhat”, 3 “strongly”, and 4 “very strongly”). The theoretical range of the scale is from 0 to 32. The higher the score is, the higher the perceived health competence. Studies using different types of samples (students, adults and persons with a chronic illness) provide evidence for the reliability and validity of the PHCS [12]. Internal consistency for our sample was α = .79.

### Data analysis

The data was organized and statistically analyzed using the Statistical Package for the Social Sciences (SPSS for Windows, version 20.0, SPSS Inc., Chicago, IL).

A General Linear Model (GLM) repeated-measures ANOVA was used to test if the outcome measures of psychosocial dental impact significantly changed over time during orthodontic treatment (baseline, at 6-month assessment and as of the end of treatment). The significance of the post hoc comparisons was calculated with the Scheffé test. All results are presented as the means (SD), and differences were considered significant at the p level < .05.

To assess the effect of the previous health competence levels (high/low) on the change from baseline to 6-month assessment, for each PIDAQ dimension, a 2*2 (time*group) repeated measures ANOVA was performed.

## Results

### Sociodemographic and orthodontic characteristics of the sample

The mean age of patients in the sample was 31.06 years (SD = 10.34; the age ranged from 18 to 62 years). Most patients were female, married and were working at the time of the study and had attended university (see Table 1).

**Table 1:** Demographic characteristics of the sample at baseline (*n* = 78)

Variable	n (%)
Sex	
Male	29 (37.17)
Female	49 (62.82)
Marital status	
Married	44 (56.41)
Single	34 (43.58)
Employment	
Employed	46 (58.97)
Unemployed	32 (41.02)
Education	
Primary School	2 (2.56)
Secondary School	22 (28.2)
University	54 (69.23)

Orthodontic treatment lasted approximately 18 months (μ = 17.25, DS = 1.29, range = 14–18). Regarding the clinical variables for the need to undergo orthodontic treatment, 93.5% of patients showed a slight treatment need (IOTN-AC ≤ 7). Only 5 patients scored IOTN-AC = 8.

### Change of the perception of psychosocial dental impact and of the IOTN-AC index during orthodontic treatment

As Table 2 shows, significant changes were observed in the variable corresponding to dental self-confidence, between T0 and T1 and between T0 and T2. In both instances, an increase in scores corresponding to dental self-confidence was observed, with no significant difference between T1 and T2.

**Table 2:** PIDAQ dimensions and IOTN-AC index before and during orthodontic treatment (*n* = 71)

	T0	T1	T2		T0/T1	T1/T2	T0/T2
	Mean (SD)	Mean (SD)	Mean (SD)	F (p)	t (p)	t (p)	t (p)
Dental self-confidence	15.64 (4.19)	21.69 (4.59)	22.61 (5.01)	13.716 (.068)	−3.858 (.001)	−.532 (.604)	−3.894 (.001)
Social impact	17.53 (6.29)	16.10 (7.05)	15.84 (6.87)	7.065 (.124)	.933 (.359)	.444 (.665)	1.409 (.189)
Psychological impact	16.28 (5.81)	13.46 (6.87)	12.30 (4.85)	1.810 (.356)	1.964 (.049)	1.080 (.301)	3.148 (.010)
Aesthetic concern	8.10 (3.21)	6.78 (3.57)	5.63 (2.87)	.380 (.725)	1.535 (.136)	.898 (.541)	2.517 (.031)
IOTN-AC	3.70 (1.94)	2.62 (1.71)	1.91 (.99)	3.750 (.211)	3.071 (.005)	.518 (.615)	7.455 (.000)

PIDAQ: Psychosocial Impact of Dental Aesthetic Questionnaire; IOTN-AC: Index of orthodontic treatment need-aesthetic concern T0: Before treatment; T1: 6 months after treatment; T2: End of treatment

The same tendency was observed regarding the psychological impact and the IOTN-AC index; significant decreases between T0 and T1 and between T0 and T2 were observed. No significant differences were observed between T1 and T2.

Finally, differences were observed between T0 and T2 for aesthetic concern.

### Health competence as a modulating variable on the change of psychosocial dental impact and the IOTN-AC index during the first 6 months of orthodontic treatment

Descriptive measures of health competence. First, a descriptive analysis of the perception of the health competence variable was performed. The data show an average score of 12.15 (SD = 5.06) with a range of scores between 4 and 23. Based on the average score of the perception of health competence, patients were classified into two groups: high (scores at or above 12.15) and low (scores below 12.15). Among the participants, 51.9% scored low on health competence, and 48.1% scored high.

The moderating effect of the health competence variable. Because the statistically significant differences observed for psychosocial dental impact and the IOTN-AC index occur between T0 and T1, the effect of health competence at T0 on the change of the PIDAQ and the IOTN-AC index was assessed between these two timepoints (T0 and T1) during the first 6 months of treatment.

As Table 3 shows, interaction effects were observed with respect to the health competence variable in the change from T0 to T1 for psychological impact, social impact, aesthetic concern and the IOTN-AC index.

**Table 3:** Factorial ANOVA (2 × 2) (time T0-T1*HC low-high) for the PIDAQ dimensions and the IOTN-AC index (*n* = 75) (only statistically significant interactions are shown).

Time (T0-T1)* group (low/high HC)	*F*	*p*	*η* ^*2*^	*Observed power*
Psychological impact	4.584	.049	.261	.509
Social impact	13.182	.003	.503	.918
Aesthetic concern	5.884	.031	.312	.612
IOTN-AC index	6.600	.025	.355	.656

T0: before treatment; T1: 6 months after treatment; HC: Heath competence; PIDAQ: Psychosocial Impact of Dental Aesthetic Questionnaire; IOTN-AC: Index of orthodontic treatment need-aesthetic concern

Specifically, for every case (social impact, psychological impact, aesthetic concern and the IOTN-AC- index) a significant favorable change was observed from T0 to T1 for patients scoring high in health competence (see Table 4). No significant differences were observed for the change from T0 to T1 for the low competence group. Additionally, no differences between high and low competence groups were observed at T0 in PIDAQ dimensions; nevertheless, at T1 (6 months later), statistically significant differences can be observed between both groups of patients in the case of social impact and psychological impact, with lower scores being observed for the high competence group.

**Table 4:** Within-group (T0-T1) and between groups (low and high HC) differences in the PIDAQ dimensions and the IOTN-AC index during the first six months of treatment (*n* = 75)

	low HC (Mean, 95%IC)	High HC (Mean, 95%IC)	Intra. Dif (T0/T1) Low high		Inter. Dif. (Low high) T0 T1	
			p	p	p	p
*Psychological impact*						
Baseline (T0)	16.00 [11.72–20.28]	17.62 [11.72–20.28]	.508	.039	.665	.099
After 6 mo. (T1)	17.29 [12.71–21.86]	11.43 [5.93–16.93]				
*Social impact*						
Baseline (T0)	16.87 [12.11–21.63]	19.86 [14.76–24.95]	.104	.005	.372	.020
After 6 mo. (T1)	20.87 [15.78–25.95]	11.71 [6.29–17.14]				
*Aesthetic concern*						
Baseline (T0)	7.87 [5.53–10.22]	7.71 [5.21–10.22]	.285	.040	.921	.008
After 6 mo. (T1)	9.25 [7.12–11.38]	4.71 [2.44–6.99]				
*IOTN-AC index*						
Baseline (T0)	2.43 [1.46–3.40]	3.86 [2.89–4.82]	.042	.814	.341	.001
After 6 mo. (T1)	2.00 [1.08–2.92]	1.86 [.94–2.77]				

T0: before treatment; T1: 6 months after treatment

HC: Health competence; PIDAQ: Psychosocial Impact of Dental Aesthetic Questionnaire; IOTN-AC: Index of orthodontic treatment need-aesthetic concern

Intra. Dif: intragroup differences; Inter. Dif: intergroup differences

Finally, regarding the IOTN-AC index, the difference in scores before treatment (T0) showing a more negative perception of malocclusion for the high competence group should be noted. No significant differences were observed at T1 for the IOTN-AC index between high and low competence groups.

## Discussion

The aim of the current study was to analyze the change of psychosocial dental impact variables and the perception of malocclusion during orthodontic treatment in a sample of adults. Additionally, the study aimed to analyze the role of patient health competence in the change of these variables.

Regarding the first aim, an interesting finding to take into account is that the same change was not observed for the different dimensions of psychosocial dental impact during treatment. In general, it seems that the first 6 months are decisive in the improvement of psychosocial dental impact. In particular, during the first 6 months of treatment, a significantly favorable change occurs in the dental impact variables associated with more personal aspects, specifically those related to emotional states and self-esteem, dental self-confidence and psychological impact. In both cases, the differences during these first 6 months remain until the end of treatment but do not increase. This change is similar to that of the patient perception of malocclusion (the IOTN-AC index).

This decrease of psychological impact along with a reduction of the self-perception IOTN-AC index can be considered a consequence of the improvement of malocclusion that occurs in the first months of treatment. This period seems to play a fundamental role in the perception of dental aesthetics, as this period is the initial phase, during which 70–100% of dental crowding is resolved [20, 21]. These findings are in agreement with those of Prado et al. who also observed such an improvement of psychological impact. Nevertheless, these authors observed no significant differences in the relationship to self-confidence between pretreatment and the first 6 months of treatment [6].

Regarding aesthetic appearance, a favorable change occurs in the long term. The study by Prado et al. obtained results in the same direction as that of the current study, suggesting a worsening of aesthetic appearance at 6 months into treatment and an improvement after the treatment has ended [6]. This improvement can be considered an effect of not only the debonding of the appliance but also the malocclusion having been corrected and an improvement of the smile [6]. Gazit-Rappaport et al. have also observed this improvement after treatment conclusion, as well as in all domains of the PIDAQ [5]. Regarding the worsening of the perception of aesthetic appearance during orthodontic treatment, other studies also observed negative effects on the quality of life of patients in the initial phases of orthodontic treatment [6]. From the first week of treatment up to the first month, the quality of life seems to worsen; this change seems to slowly improve over time until the end of treatment and could be explained by the discomfort and initial pain caused by the appliance [7, 22]. This worsening of QoL has also been observed to persist even up to the first 3 months of treatment, returning to baseline values at debonding [4, 23].

The above results seem to apply regardless of the type of brackets used. Previous studies show, in general, an absence of significant improvements in QoL regardless of whether conventional or self-ligating brackets are used. The only differences seem to be in relation to physical pain, which is lower for self-ligating brackets, although the differences are not statistically significant [10].

Finally, the social impact does not seem to change during treatment. A possible explanation for this could be that orthodontic treatment has been normalized within present society, as it is increasingly frequent for adults to undergo treatment and doing so is considered an attempt to improve the oral health and the aesthetics of the smile [24–26].

As to the second aim, the results show that the patient perception of health competence plays a fundamental role in the changeof psychosocial dental impact variables during orthodontic treatment. Health competence has been studied in relation to better compliance with treatment in chronic diseases and physical activity [27, 28]. A concept that is close to health competence is self-efficacy (Bandura, 1977), although some authors prefer health competence, as self-efficacy is a wider concept that also applies to other areas, and therefore, there is a stronger predictive value when perception of competence refers to the subject related to behavior, in this case, health [12, 29]. Although studies are scarce, the concept of self-efficacy has been studied in relation to odontology; however, to our knowledge, there are no studies related to health competence. Self-efficacy has predicted a range of health behaviors, including oral self-care; however, few studies have associated self-efficacy with dental practice [30, 31]. Among such studies, the majority of published studies of self-efficacy in the dental field have focused on how it affects the performance of oral hygiene behaviors, especially in the periodontal areas [32, 33].

In terms of orthodontic treatment, there are few studies analyzing the role of self- efficacy, but these studies have shown that this variable contributes to the differences between patients with high and low positive affect 6 months after orthodontic treatment [34]. Other longitudinal studies show that a lack of orthodontic treatment while the patients were teenagers, where there was a prior need, does not lead to psychological difficulties in later life [35]. Dental status alone is a weak predictor of self-esteem in adulthood, and it is self-efficacy that has one of the strongest predictive values in this sense [35, 36].

The results show, in accordance with the findings of other authors, the importance of health competence as a key variable in the improvement the psychosocial dental impact of patients during the first 6 months of orthodontic treatment. This result is especially significant for the self-perception of malocclusion and the (IOTN-AC) index, as the group with high competence, despite starting off with a significantly worse self-perception, obtained better scores on this index 6 months later than did the low competence group.

The primary limitation of this study is the convenient nature and the sample size (78 patients), composed of voluntary patients recruited through a University Clinic [omitted for blind review]; this aspect of the sample may limit its representativeness and, thus, prevent the generalization of the findings of this study to the general population. The second limitation of the article is the severity of the malocclusion. We chose mild or moderate malocclusions because they are most frequently observed in the dental clinic. In future research, we will expand our sample to observe changes based on different degrees of malocclusion and increase the sample size.

Nevertheless, despite the above limitation, this study should be considered to have important practical repercussions regarding orthodontic treatment.

## Conclusions

1. Change in the perception of the psychosocial dental impact of orthodontic treatment (in mild/moderate dental malocclusions) depends on the dimension of dental impact.

2. The first 6 months of treatment are key to the improvement in psychological subscales (self-confidence and psychological impact) in the perception of dental impact.

3. An improvement in aesthetic concerns does not occur until the end of treatment.

4. The social impact dimension is not altered throughout the treatment.

5. The perception of health competence plays a fundamental role in the improvement of all indicators regarding psychosocial dental impact in the short term (the first 6 months of treatment).

The results have important practical repercussions. On the one hand, the results are relevant to the consideration of dental impact indicators within biopsychosocial models throughout the orthodontic process. These indicators should always be included in the orthodontic check-up.

On the other hand, the results relate to how the psychosocial dental impact subscales varies throughout the treatment process. Thus, dental self-confidence and psychological impact constitute valid indicators of dental treatment and dental impact in the short term, while the aesthetic concern is a reliable long-term indicator. They are, in short, indicators of the impact of treatment, at different temporal moments, on the patient quality of life. Finally, the perception of health competence should be initially evaluated as a key variable for the improvement of psychosocial dental impact within the first 6 months of treatment. In addition, this variable should be considered in every patient’s initial assessment as a way to improve patient adherence to treatment.

## Data Availability

Not applicable.

## Abbreviations

IOTN-AC: Aesthetic component of the IOTN index
OHRQoL: Oral health-related quality of life
PHCS: Perceived Health Competence Scale
PIDAQ PIDAQ: Psychosocial Impact of Dental Aesthetics
QoL: Quality of life
